# Phylogenomic and comparative genomic analyses of *Aeromonas* spp. from South American aquatic systems reveal extensive genomic diversity, antimicrobial resistance, and predicted human pathogenicity

**DOI:** 10.3389/fmicb.2026.1823138

**Published:** 2026-06-16

**Authors:** Enrique Garcia-Candela, Aarón Mondragón-Martínez, Gerald Moreno-Morales, Milagros Cabrera-Soregui, Víctor Humberto Puicón Niño de Guzmán, Juan C. Ramos Gorbena, Alcides Guerra Santa Cruz, Lidia Cruz-Neyra, Julio Solis-Sarmiento

**Affiliations:** 1 CITE productivo San Martín, Instituto Tecnológico de la Producción, San Martín, Peru; 2 Grupo de Investigación en Ciencias Ómicas y Bioinformática (GICOB), Facultad de Medicina Veterinaria, Universidad Nacional de San Martín, Tarapoto, Perú; 3 Laboratorio de Biología Molecular y Genómica, Escuela profesional de Biología, Facultad de Ciencias Biológicas, Universidad Ricardo Palma (URP), Peru; 4 Instituto de Calidad, Inocuidad y Seguridad Alimentaria, Universidad Ricardo Palma (URP), Lima, Peru; 5 Unidad de Biología y Genética Molecular, Facultad de Medicina Veterinaria, Universidad Nacional Mayor de San Marcos (UNMSM), Lima, Peru; 6 Grupo de investigación en Parasitología Veterinaria y Zoonosis Parasitaria, Facultad de Medicina Veterinaria, Universidad Nacional de San Martín, Tarapoto, Peru; 7 Laboratorio de Genómica y Bioinformática Aplicada (Lab-Bio-Gen), Facultad de Ciencias Biológicas, Universidad Nacional Mayor de San Marcos (UNMSM), Lima, Peru

**Keywords:** *Aeromonas*, AMR (antimicrobial resistance), aquatic system, genomic, phylogenomic analyses

## Abstract

**Introduction:**

Aquaculture systems function as dynamic ecological interfaces facilitating the circulation of opportunistic pathogens and antimicrobial resistance (AMR) determinants across environmental, animal, and human compartments. *Aeromonas* spp. are ubiquitous freshwater bacteria and emerging human opportunistic pathogens, yet genomic data from tropical South American aquaculture remain scarce.

**Methods:**

Nine *Aeromonas* isolates from Amazonian aquaculture facilities in Peru were subjected to whole-genome sequencing. Species identification was performed using Average Nucleotide Identity and phylogenomic reconstruction within a dataset of 112 genomes. Ribosomal MLST, comparative gene content analysis, virulence and resistome profiling, human pathogenicity prediction, and quinolone resistance-determining region (QRDR) screening were conducted. Phenotypic antimicrobial susceptibility testing was performed against five antibiotics.

**Results:**

Phylogenomic analysis revealed substantial genetic diversity among the analyzed isolates, encompassing *Aeromonas hydrophila, Aeromonas caviae*, and *Aeromonas veronii*, with no clustering by host species or production site. Ribosomal MLST assigned 8 distinct rSTs across the 9 isolates. Comparative genomic analysis identified 19,486 gene clusters, of which a subset was shared across all genomes, while the majority were variably distributed, indicating extensive genomic diversity across *Aeromonas* spp. All isolates shared conserved colonization- and secretion-associated determinants, whereas major cytotoxic toxin genes were absent. PathogenFinder v2 predicted high probabilities of human pathogenicity (0.93–0.96) across all genomes. Resistome analysis identified genes associated with tetracycline, quinolone, sulfonamide, and β-lactam resistance, including intrinsic blaOXA and cphA, while QRDR screening identified mutations potentially associated with quinolone resistance. Genomic findings were broadly consistent with phenotypic susceptibility profiles.

**Conclusion:**

Amazonian aquaculture systems harbor genetically diverse *Aeromonas* lineages with conserved opportunistic virulence traits and clinically relevant AMR determinants. These findings highlight the role of aquaculture environments as reservoirs and potential transmission interfaces of antimicrobial resistance and opportunistic pathogens, underscoring the importance of genomic surveillance within a One Health context.

## 1 Introduction

Global aquaculture has surpassed capture fisheries since 2022, now providing 98.5 million tons (52%) of the 189 million tons of aquatic animals produced globally, compared to 90 million tons (48%) from wild capture (FAO, 2025). This rapid intensification has consolidated aquaculture as the primary source of aquatic protein worldwide and a strategic sector for global food security. However, production intensification has paradoxically increased vulnerability to infectious diseases, with bacterial pathogens—particularly *Aeromonas* spp.—representing the principal sanitary constraint in freshwater aquaculture systems.

The genus *Aeromonas* comprises principal bacterial pathogens in global freshwater aquaculture, causing motile aeromonad septicemia documented across Asia and Latin America, resulting in mass mortality events and substantial economic losses in cultured tilapia, catfish, and native Amazonian species (Pattanayak et al., 2020; Semwal et al., 2023). Beyond aquaculture, *Aeromonas* species function as opportunistic zoonotic organisms capable of infecting humans and terrestrial animals through water exposure, handling of infected fish, or consumption of undercooked aquatic products. In humans, infections range from gastroenteritis to soft tissue infections and septicemia, particularly in immunocompromised individuals (Syanya et al., 2025). This dual ecological and pathogenic role positions *Aeromonas* at the human–animal–environment interface, making it fundamentally relevant within the One Health framework.

The pathogenicity of *Aeromonas* is supported by a complex arsenal of virulence determinants, including type II, III, and VI secretion systems involved in toxin delivery, type IV pili mediating adhesion, and hemolysins capable of disrupting host cell membranes and inducing hemorrhagic manifestations (Tomás, 2012). However, virulence is not uniformly distributed among strains. Instead, the genus exhibits remarkable genomic diversity, where a relatively small proportion of genes is conserved across all strains, while a large fraction corresponds to accessory genes variably distributed among lineages (Ghatak et al., 2016; Abdella et al., 2023). This genomic variability enables individual lineages to acquire unique combinations of virulence and adaptive traits, particularly through horizontal gene transfer and recombination, thereby explaining observed differences in pathogenic potential and host range across strains (Khor et al., 2015; Islam et al., 2018).

The intensification of aquaculture has been accompanied by extensive antimicrobial use, with global consumption exceeding 10,000 tons annually (Milijasevic et al., 2024). This selective pressure has transformed aquatic environments into reservoirs of multidrug-resistant bacteria, with *Aeromonas* species frequently harboring resistance determinants against β-lactams, quinolones, aminoglycosides, and sulfonamides—often encoded on mobile genetic elements that facilitate dissemination across aquatic and terrestrial ecosystems (Hossain et al., 2022; Caputo et al., 2023; Suyamud et al., 2024). Notably, antimicrobial resistance profiles exhibit substantial heterogeneity among *Aeromonas* strains, reflecting lineage-specific acquisition and dissemination of resistance mechanisms (Islam et al., 2018).

The Peruvian Amazon represents a region of particular sanitary and economic relevance, where the San Martín region supports freshwater aquaculture based on native Amazonian species such as paiche (*Arapaima gigas*), one of the largest freshwater fish species worldwide; gamitana (*Colossoma macropomum*), a robust species adapted to hydrological fluctuations; and paco (*Piaractus brachypomus*), valued for its resilience and commercial importance (Dávila et al., 2022; Hilsdorf et al., 2022). These production systems coexist with Nile tilapia (*Oreochromis niloticus*), an introduced species that dominates regional aquaculture due to its high productivity and adaptability (Haenen et al., 2023). Despite documented *Aeromonas*-associated disease outbreaks causing mass mortality in Amazonian aquaculture—particularly in native species such as *P. brachypomus* and *A. gigas*—genomic characterization of regional strains has remained limited to conventional approaches. Published studies have primarily employed partial 16S rRNA sequencing and PCR-based detection of selected virulence markers (Moya-Salazar et al., 2022; Medina-Morillo et al., 2023), with scarce whole-genome sequencing data available to assess antimicrobial resistance profiles and phylogenomic relationships within this strategically important aquaculture region.

Whole-genome sequencing has revolutionized the study of aquatic pathogens by enabling the reconstruction of evolutionary histories, comprehensive identification of virulence and antimicrobial resistance repertoires, inference of global phylogenetic relationships, and tracking of pathogenic lineage dissemination (Bayliss et al., 2017; Lee et al., 2019; Bartie et al., 2023; Singh et al., 2026). Within this framework, comparative genomic approaches enable the characterization of gene content diversity by distinguishing genes conserved across all strains from those variably distributed among subsets of isolates, which are frequently associated with virulence, antimicrobial resistance, and ecological adaptation (Tettelin et al., 2005; Rouli et al., 2015).

In *Aeromonas*, variation in accessory gene content plays a key role in explaining clinically relevant differences in virulence and antimicrobial resistance profiles among strains. While certain systems, such as type II and type VI secretion systems, are broadly conserved, other determinants—including type III secretion systems—exhibit substantial variability across lineages (Islam et al., 2018). This genomic heterogeneity provides a framework to understand why phenotypically similar *Aeromonas* isolates may display distinct pathogenic behaviors. To address this knowledge gap, this study presents the first whole-genome sequencing-based characterization of *Aeromonas* spp. circulating in Amazonian freshwater aquaculture. Our objectives were to: (1) systematically characterize the virulence repertoires and antimicrobial resistance determinants of *Aeromonas* isolates recovered from both native and introduced fish species in the San Martín region; (2) infer phylogenomic relationships and population structure through core-genome alignment and multilocus sequence typing; and (3) contextualize these isolates within a broader South American genomic framework by comparison with publicly available genomes derived from diverse ecological sources. By integrating whole-genome sequencing, phylogenomics, comparative genomics, and phenotypic antimicrobial susceptibility testing within a One Health framework, this work provides a foundational platform for molecular surveillance, prediction of pathogenic potential in emerging lineages, and the development of ecosystem-adapted disease management strategies for Amazonian aquaculture systems.

## 2 Material and methods

### 2.1 Bacterial strain collection

A total of nine presumptive *Aeromonas* spp. isolates were obtained from the bacterial collection of the Laboratory for Research and Analysis of Aquaculture Health, CITEacuícola (LIA-CITE, Tarapoto, Peru). The isolates were recovered from tropical freshwater fish during disease outbreaks and routine health monitoring conducted between 2021 and 2022 at aquaculture facilities located in the Amazonian regions of San Martín and Ucayali, Peru.

### 2.2 Antimicrobial susceptibility testing (AST)

Antimicrobial susceptibility of the isolates was evaluated using the disk diffusion method in accordance with the guidelines of the Clinical and Laboratory Standards Institute (CLSI). The following commercial antibiotic disks were tested: oxytetracycline (OT, 30 μg), oxolinic acid (OA, 2 μg), florfenicol (FFC, 30 μg), enrofloxacin (ENR, 5 μg), and trimethoprim–sulfamethoxazole (SXT, 25 μg). All antibiotic disks were obtained from Oxoid Ltd. (Basingstoke, Hampshire, UK).

Bacterial suspensions were prepared in phosphate-buffered saline (PBS) and adjusted to a turbidity equivalent to a 0.5 McFarland standard, followed by inoculation onto Mueller–Hinton agar plates (MHA; HiMedia, India). Plates were incubated at 28 °C for 24–28 h. *Escherichia coli* ATCC 25922 was included as a quality control strain. Inhibition zone diameters (IZDs) were measured, and isolates were classified as susceptible (S), intermediate (I), or resistant (R) according to CLSI and EUCAST interpretative criteria (Clinical and Laboratory Standards Institute (CLSI), 2018; European Committee on Antimicrobial Susceptibility Testing (EUCAST), 2018).

### 2.3 Whole-genome sequencing, assembly, annotation, and genome-based identification

Genomic DNA was extracted using the Wizard^®^ Genomic DNA Purification Kit (Promega, Madison, WI, USA). DNA quantity and quality were assessed using a Quantus™ Fluorometer with the QuantiFluor™ dsDNA System (Promega) and by electrophoresis on 1% agarose gels. Sequencing libraries were prepared using the TruSeq Nano DNA Fragment Library Preparation Kit (Illumina, USA) and sequenced on an Illumina NovaSeq 6000 platform to generate 150 bp paired-end reads.

Raw sequencing reads were assessed for quality using FastQC v0.11.9 (Andrews, 2010). Adapter sequences and low-quality bases (*Q* < 30) were trimmed using Trimmomatic v0.39 (Bolger et al., 2014). High-quality reads were *de novo* assembled using Unicycler v0.5.1 (Wick et al., 2017). Assembly quality metrics were evaluated using QUAST (Gurevich et al., 2013), and genome completeness and contamination were assessed using CheckM v1.2.3 (Parks et al., 2015).

Genome annotation was performed using Prokka (Seemann, 2014). Circular genome maps were generated using the BLAST Ring Image Generator (BRIG) (Alikhan et al., 2011).

Genome-based species identification was performed using Average Nucleotide Identity (ANI) calculated with FastANI v1.34 (Jain et al., 2018). Each genome was compared against reference genomes retrieved from the BV-BRC database, including type and representative genomes of *Aeromonas* species. Reference genomes were selected based on high assembly quality (completeness ≥95% and contamination ≤ 5%), as estimated using CheckM, and taxonomic reliability. Species assignments were determined using a ≥95% ANI threshold. Genome metadata and assembly quality metrics are provided in Supplementary Table S1. Pairwise ANI comparisons across all genomes and within-species subsets are reported in Supplementary Tables 2, 3.

### 2.4 Genome dataset for comparative genomic analysis

A total of 112 publicly available *Aeromonas* genomes were retrieved from the BV-BRC database for comparative genomic and phylogenomic analyses. Genome selection was based on assembly quality and representativeness criteria. Only genomes with high completeness (≥95%) and low contamination (≤5%), as assessed by CheckM, were included. To ensure a representative dataset, genomes from multiple *Aeromonas* species, geographic regions, isolation sources, and sampling years were considered (see Supplementary Table 1). In particular, genomes were selected to reflect a One Health framework, with particular emphasis on South American isolates to contextualize the regional epidemiology of *Aeromonas* spp.

### 2.5 Multilocus sequence typing (MLST)

Multilocus sequence typing (MLST) was performed *in silico* using genome assemblies of the nine *Aeromonas* isolates. Ribosomal sequence types (rSTs) were assigned using the ribosomal MLST (rMLST) scheme, which is based on 53 conserved genes encoding ribosomal protein subunits. Sequences were queried against the rMLST database available at PubMLST (https://pubmlst.org/species-id) using the Sequence Query tool. The resulting allelic profiles were used to assign rSTs and to assess the clonal relationships among isolates. Species-level identification was primarily determined based on Average Nucleotide Identity (ANI), while rMLST results were used as complementary evidence for phylogenetic consistency (Jolley et al., 2018).

### 2.6 Phylogenomic Reconstruction and Comparative Gene Content Analysis in *Aeromonas* spp.

A phylogenomic analysis was performed using a dataset of 112 *Aeromonas* genomes, including nine genomes generated in this study. Publicly available genomes were retrieved from the BV-BRC database and selected based on assembly quality and representativeness criteria, including high completeness (≥95%) and low contamination (≤5%), as assessed using CheckM. To ensure a representative dataset, genomes from multiple *Aeromonas* species, geographic regions, isolation sources, and sampling years were included, following a One Health framework.

Core genome identification and alignment were carried out using Panaroo v1.6.0 (https://github.com/gtonkinhill/panaroo) in strict mode to minimize paralogous genes and annotation artifacts. Core genes were defined following Panaroo criteria (≥99% genome presence). The resulting core genome alignment comprised 2,223 genes shared across all genomes in the dataset, corresponding to a total alignment length of 805,418 bp. To account for the impact of homologous recombination on phylogenetic inference, the alignment was further processed using Gubbins v3.4.3 (Croucher et al., 2015), which identifies and removes recombinant regions to generate a recombination-filtered alignment containing only vertically inherited polymorphic sites.

Maximum-likelihood phylogenetic inference was conducted using RAxML-NG v1.2.2 (Kozlov et al., 2019) under a GTR+G substitution model. Phylogenetic reconstruction was performed using the recombination-filtered alignment generated by Gubbins. Empirical nucleotide frequencies and among-site rate heterogeneity were estimated during tree inference, with rate variation modeled using a gamma distribution. Branch support was assessed using 1,000 bootstrap replicates. To characterize gene content variation across the dataset, the Panaroo gene presence–absence matrix, comprising 19,486 gene clusters, was analyzed using custom Python scripts. This enabled the identification of genes shared across all genomes in the dataset and those variably distributed, allowing the exploration of gene content patterns in relation to phylogenomic structure, without inferring pangenome openness or structure. All phylogenomic trees and gene presence–absence datasets generated in this study are provided in the Supplementary Material and have been deposited in a public repository (Zenodo) under doi: 10.5281/zenodo.19989926, ensuring reproducibility and long-term accessibility.

### 2.7 *In silico* identification of virulence and antimicrobial resistance genes

Virulence-associated genes were identified using VFAnalyzer based on the Virulence Factor Database (VFDB) (Liu et al., 2022), applying a minimum sequence identity threshold of 80% and a minimum coverage of 70%. In parallel, additional screening of virulence and antimicrobial resistance genes was performed using ABRicate v1.0.1 (https://github.com/tseemann/abricate) across multiple curated databases, applying thresholds of ≥80% sequence identity and ≥70% coverage to consider a gene as present. The presence of antimicrobial resistance (AMR) genes was predicted using NCBI AMRFinderPlus v4.2.7 (Feldgarden et al., 2021) and ResFinder v4.7.0 (Bortolaia et al., 2020). AMRFinderPlus was run using default parameters, which include curated thresholds for gene detection, while ResFinder analysis applied a minimum sequence identity threshold of 90% and minimum coverage of 60%. Results from the different tools were cross-validated to increase confidence in gene detection and annotation.

### 2.8 Prediction of human pathogenic potential

Prediction of human pathogenic potential was performed using PathogenFinder v2 (Ferrer Florensa et al., 2025), a web-based tool provided by the Center for Genomic Epidemiology, for all nine Amazonian *Aeromonas* genomes analyzed.

## 3 Results

### 3.1 Isolate origin and genome-based species identification

**Figure 1:**
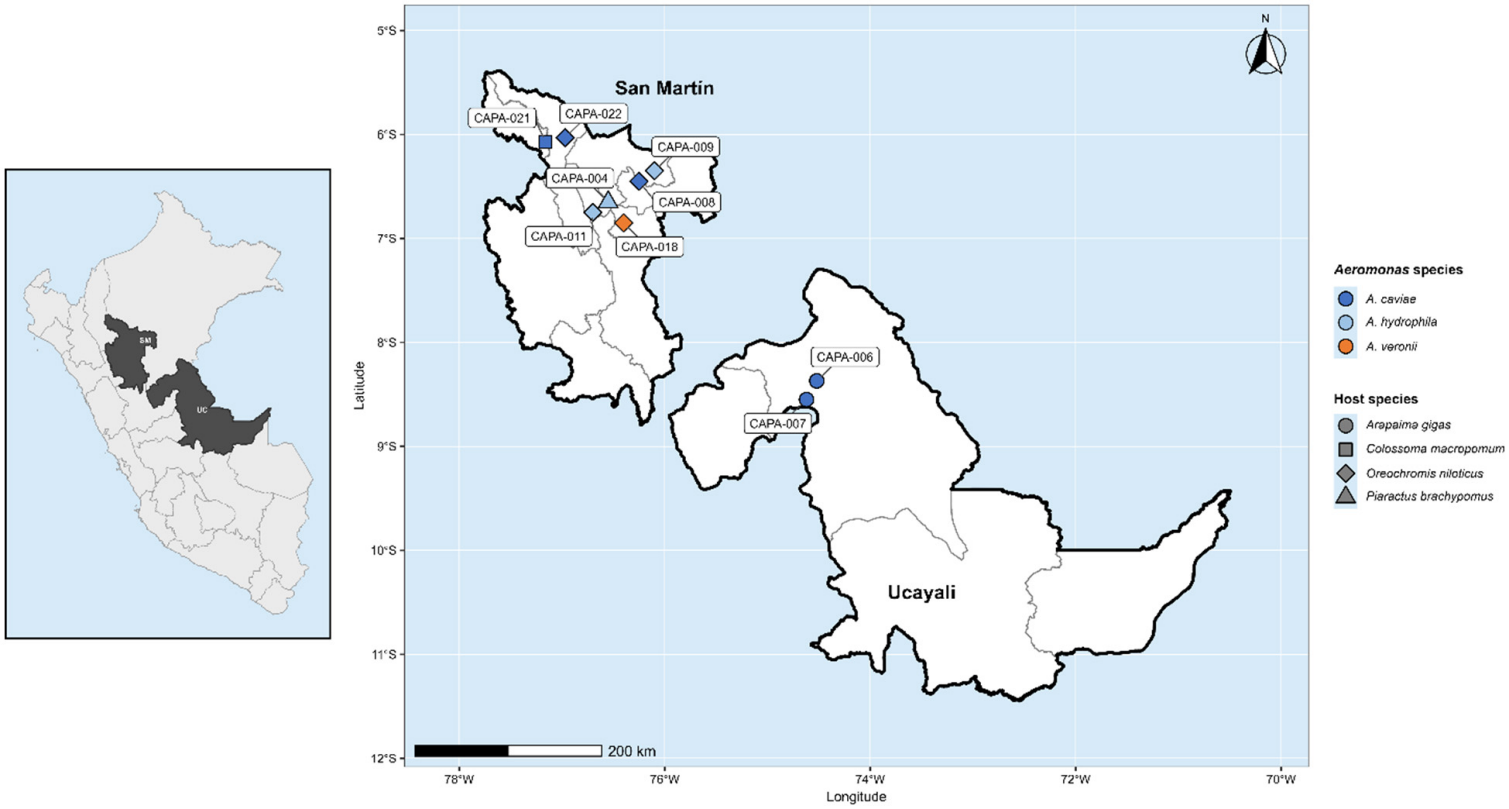
Geographic distribution of *Aeromonas* isolates recovered from freshwater aquaculture facilities in the San Martín and Ucayali regions, Peru (2021–2022). The inset map indicates the location of the sampled regions within Peru (dark gray). Symbol shape represents host fish species, and symbol color indicates *Aeromonas* species assignment based on Average Nucleotide Identity (ANI) analysis. Strain identifiers are shown for each sampling location.

Nine *Aeromonas* isolates were analyzed in this study, recovered from tropical freshwater fish during disease outbreaks and health monitoring activities conducted between 2021 and 2022 in aquaculture facilities located in the Amazonian regions of San Martín and Ucayali, Peru. The isolates originated from four fish species, including Nile tilapia (*Oreochromis niloticus*), paiche (*Arapaima gigas*), gamitana (*Colossoma macropomum*), and pacu (*Piaractus brachypomus*) (Figure 1).

Genome-based species identification using Average Nucleotide Identity (ANI) confirmed that all isolates belonged to the genus *Aeromonas*, with values above the accepted species-level threshold (≥95%) when compared to reference genomes. The isolates were assigned to three species: *Aeromonas hydrophila* (*n* = 3), *Aeromonas caviae* (*n* = 5), and *Aeromonas veronii* (*n* = 1), revealing species-level diversity among isolates recovered from different hosts and geographic locations. These assignments were further supported by phylogenomic clustering and *in silico* multilocus sequence typing (MLST), which showed consistent grouping across classification approaches. Detailed ANI results are provided in Supplementary Figure 1 and Supplementary Tables S2–S3.

### 3.2 Antimicrobial susceptibility profiles of *Aeromonas* isolates

Phenotypic antimicrobial susceptibility testing revealed heterogeneous resistance profiles among the nine *Aeromonas* isolates. Susceptibility results for the five antibiotics tested are summarized in Table 1. Resistance was most frequently observed for oxytetracycline (77.7%, *n* = 7), followed by oxolinic acid (55.5%, *n* = 5). Lower resistance frequencies were observed for enrofloxacin and sulfamethoxazole/trimethoprim, while florfenicol resistance was limited to a single isolate. Multidrug resistance (MDR), defined as resistance to ≥3 antimicrobial classes, was detected in four isolates (44.4%), predominantly among A. caviae strains. Overall, the observed resistance patterns indicate variability in antimicrobial susceptibility across isolates, with a subset exhibiting clinically relevant multidrug resistance profiles.

**Table 1:** Antimicrobial susceptibility profiles of nine *Aeromonas* isolates from Amazonian aquaculture systems in Peru, determined by disk diffusion method according to CLSI guidelines.

Isolate	Species	Resistance pattern	No. resistant antibiotics
CAPA004	*Aeromona hydrophila*	OT, FFC, OA, SXT, ENR	5
CAPA006	*Aeromona caviae*	OT, OA, SXT, ENR	4
CAPA007	*Aeromona caviae*	OT, OA, SXT, ENR	4
CAPA008	*Aeromona caviae*	OT, OA, SXT	3
CAPA009	*Aeromona hydrophila*	OT	1
CAPA011	*Aeromona hydrophila*	NA	0
CAPA018	*Aeromona veronii*	OT, OA	2
CAPA021	*Aeromona caviae*	OT	1
CAPA022	*Aeromona caviae*	—	—

OT, oxytetracycline; OA, oxolinic acid; FFC, florfenicol; ENR, enrofloxacin; SXT, trimethoprim–sulfamethoxazole; NA, no resistance detected. Resistance patterns were determined based on inhibition zone diameter interpretive criteria established by Clinical and Laboratory Standards Institute (CLSI) (2018) and European Committee on Antimicrobial Susceptibility Testing (EUCAST) (2018).

Analysis of individual resistance patterns showed that most isolates exhibited resistance to at least one antimicrobial agent. Multidrug resistance (MDR), defined as resistance to three or more antimicrobial classes, was identified in isolates belonging to *Aeromonas hydrophila* and *A. caviae*, whereas two isolates (*A. hydrophila* CAPA011 and *A. caviae* CAPA021) were susceptible to all tested antibiotics.

Among *A. hydrophila* isolates (*n* = 3), resistance to oxytetracycline was observed in two isolates, while susceptibility to florfenicol, enrofloxacin, trimethoprim–sulfamethoxazole, and oxolinic acid predominated. The single *A. veronii* isolate exhibited resistance to oxytetracycline and oxolinic acid, while remaining susceptible to florfenicol, enrofloxacin, and trimethoprim–sulfamethoxazole. Among *A. caviae* isolates (*n* = 5), resistance to oxytetracycline was detected in four isolates and to oxolinic acid in three, whereas all isolates were susceptible to florfenicol.

Isolates of *A. hydrophila* and *A. caviae* exhibiting multidrug-resistant phenotypes showed elevated MAR values, whereas the *A. veronii* isolate presented a lower MAR index and did not meet MDR criteria.

### 3.3 Genome sequencing, assembly quality, and species-level assignment

**Figure 2:**
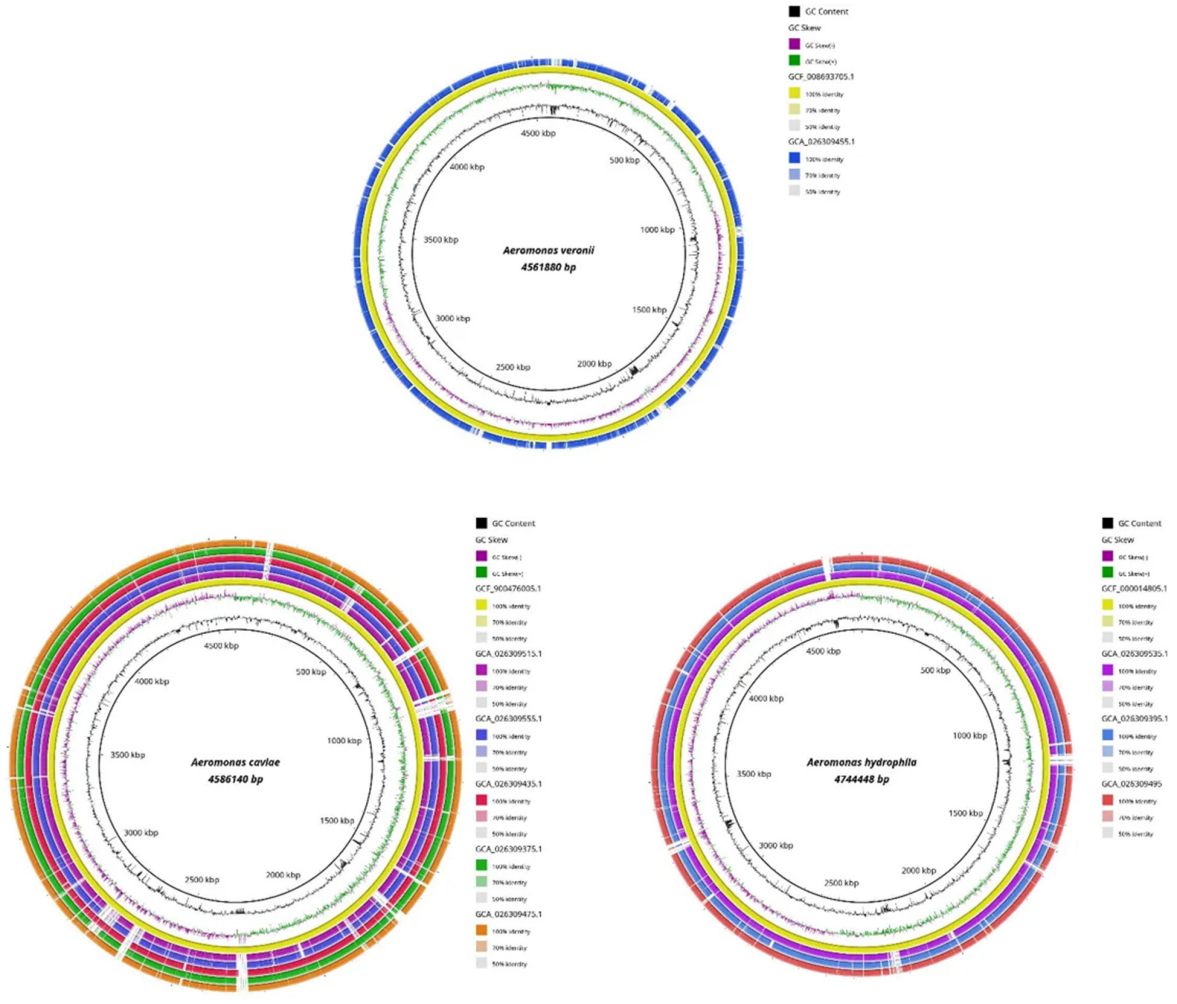
Circular genome maps of nine *Aeromonas* isolates recovered from Amazonian aquaculture systems in Peru, generated using the BLAST Ring Image Generator (BRIG). Each ring represents a genome comparison against a reference genome (*A. hydrophila* GCF_000014805.1, *A. caviae* GCF_900476005.1, and *A. veronii* GCF_008693705.1). GC content and GC skew are displayed in the inner rings. Genome size and GC content (%) are indicated for each isolate.

Whole-genome sequencing of the nine *Aeromonas* isolates generated high-quality draft genome assemblies, with an average sequencing depth of approximately 150×. Genome sizes ranged from 4.40 to 5.21 Mb, and GC contents (58.5–61.7%) were consistent with those reported for the genus *Aeromonas* (Figure 2). The assemblies comprised between 28 and 166 contigs, with N50 values ranging from 106,306 to 912,620 bp.

Genome quality assessment indicated excellent assembly completeness, with CheckM estimates ranging from 99.84% to 100% and no detectable contamination. The number of predicted coding sequences varied from 4,221 to 5,053 CDSs, along with 91–99 tRNA genes and 3–5 rRNA operons per genome.

To contextualize the study isolates within a broader phylogenomic and comparative framework, an expanded dataset comprising 112 *Aeromonas* genomes was assembled. Publicly available genomes were retrieved from the BV-BRC database and filtered using stringent quality criteria, retaining only assemblies classified as “Good” (see Supplementary Table 1). The selected genomes exhibited high completeness (≥97.36%) and low contamination (≤4.95%), ensuring their suitability for downstream phylogenomic reconstruction and comparative genomic analyses.

### 3.4 Multilocus sequence typing and clonal diversity

*In silico* multilocus sequence typing (MLST) analysis of the nine *Aeromonas* genomes revealed a high level of genetic diversity. Ribosomal MLST analysis assigned rSTs to all nine *Aeromonas* isolates. Among *A. caviae* isolates, CAPA008 and CAPA022 shared rST 220374, while CAPA021, CAPA007, and CAPA006 were assigned to rST 220373, rST 220372, and rST 220371, respectively. Among *A. hydrophila* isolates, CAPA004, CAPA009, and CAPA011 were assigned to rST 97532, rST 121722, and rST 26584, respectively. The single *A. veronii* isolate (CAPA018) was assigned to rST 220400. The detection of eight distinct rSTs across nine isolates confirms a multiclonal population structure. Notably, CAPA008 and CAPA022 shared an identical rST, suggesting potential clonal relatedness despite their recovery from independent aquaculture systems. All rST assignments were concordant with genome-based species identification.

### 3.5 Phylogenomic structure and comparative gene content of *Aeromonas* spp.

**Figure 3:**
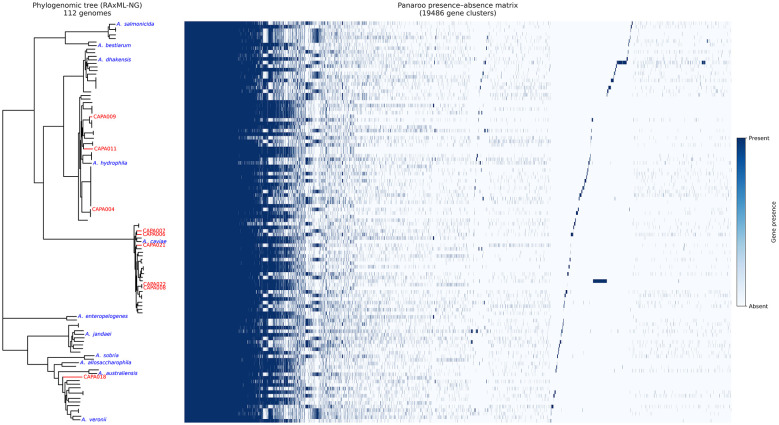
Phylogenomic reconstruction and gene content variation across 112 *Aeromonas* genomes. The maximum-likelihood phylogenetic tree **(left)** was inferred using RAxML-NG based on a recombination-filtered alignment of genes shared across the dataset. The inferred topology is supported by high bootstrap values. Study isolates are highlighted in red, and reference genomes are indicated in blue. The heatmap **(right)** represents the gene presence–absence matrix comprising 19,486 gene clusters, illustrating patterns of gene content variation across species and intra-species lineages.

Phylogenomic reconstruction based on the core genome alignment resolved the 112 *Aeromonas* genomes into multiple well-supported clades corresponding to recognized species, each forming monophyletic groups with strong bootstrap support (>90%) (Figure 3). The inclusion of reference genomes representing distinct *Aeromonas* species enabled accurate species-level placement of the study isolates and confirmed their taxonomic assignments inferred from genome-based identification.

Within-species phylogenetic structure revealed substantial intra-species diversity, with the study isolates distributed across distinct, well-supported subclades. Among *A. caviae* isolates, CAPA022 and CAPA008 clustered within the same lineage, whereas CAPA021 and CAPA007 formed separate lineages within the species clade. CAPA006 grouped closely with a reference *A. caviae* genome, suggesting phylogenetic proximity to previously described strains.

The *A. hydrophila* clade exhibited pronounced internal structure, with CAPA011, CAPA009, and CAPA004 each occupying distinct phylogenetic positions supported by high bootstrap values, indicative of considerable genomic diversity among isolates. In contrast, the *A. veronii* clade contained a single study isolate (CAPA018), which formed a well-supported lineage within the species cluster.

Notably, isolates originating from different sources and hosts were interspersed within species-level clades, rather than forming source-specific clusters. This pattern supports a One Health perspective and suggests that closely related *Aeromonas* lineages may circulate across environmental, animal, and human-associated niches in South America.

To assess the impact of homologous recombination on phylogenetic inference, core genome alignments were further processed using Gubbins. The resulting recombination-filtered phylogeny showed a topology highly consistent with the original tree, with no major changes in species clustering or lineage relationships. This consistency indicates that the inferred population structure is robust and largely driven by vertically inherited genetic variation, with limited influence of homologous recombination on the overall phylogenetic signal.

Comparative analysis of gene content across the 112 *Aeromonas* genomes identified a total of 19,486 gene clusters. Of these, 2,223 genes were shared across all genomes in the dataset under the applied threshold (≥99% genome presence), while the remaining genes were variably distributed among isolates, reflecting substantial genomic diversity. The distribution of gene content across species and intra-species lineages highlights the heterogeneous genomic composition of *Aeromonas* populations. Visualization of the gene presence–absence matrix in the context of the phylogenomic tree revealed patterns of gene content variation associated with phylogenetic structure, providing genomic context for the lineage differentiation observed in the phylogenetic reconstruction (Figure 3).

These results are interpreted as a comparative framework of gene content variation rather than a formal pangenome characterization.

### 3.6 Distribution of virulence-associated genes

*In silico* screening of virulence-associated genes revealed a largely conserved virulence gene repertoire across the nine *Aeromonas* genomes analyzed. Genes associated with motility and host colonization were consistently detected in all isolates (9/9), including the complete flagellar gene cluster (*flaA–flaE*), type IV pilus-associated genes (*pilT* and *pilU*), and the msh operon encoding the mannose-sensitive hemagglutinin (MSHA) pilus.

Genes involved in secretion and host interaction were also universally present. These included components of the type II secretion system (T2SS), such as *xcpR* and *gspD*, which are responsible for the secretion of extracellular enzymes and proteins involved in host interactions. In addition, all genomes harbored *luxS*, a key gene involved in quorum sensing. Genes associated with structural components related to host interaction were detected across the dataset, including *kdsA*, which is involved in lipopolysaccharide (LPS) biosynthesis.

**Figure 4:**
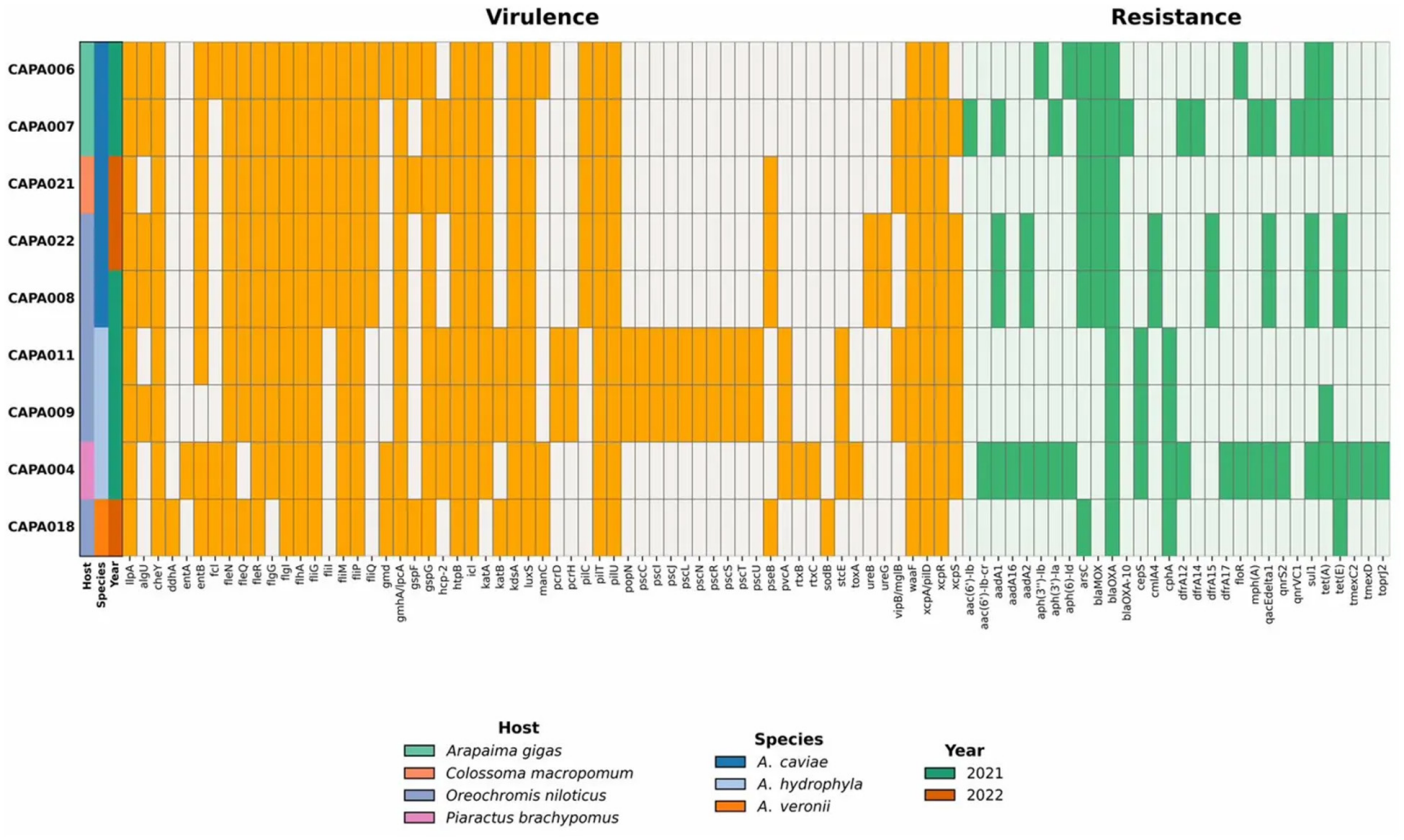
Distribution of virulence-associated and antimicrobial resistance genes among nine *Aeromonas* isolates from Amazonian aquaculture systems in Peru. The heatmap displays presence (dark) and absence (white) of genes identified through *in silico* screening using VFDB, ABRicate, AMRFinderPlus, and ResFinder. Isolates are grouped by species and genes are organized by functional category.

In contrast, major cytotoxic and enterotoxin genes commonly associated with highly virulent *Aeromonas* strains, including *aerA* (aerolysin), act (cytotoxic enterotoxin), and *ast* (heat-stable enterotoxin), were not detected in any of the analyzed genomes (Figure 4).

### 3.7 Distribution of antimicrobial resistance genes

*In silico* resistome analysis of the nine *Aeromonas* genomes revealed the presence of multiple antimicrobial resistance (AMR) genes associated with compounds used in aquaculture, veterinary medicine, and human clinical settings.

Resistance genes related to antimicrobials commonly applied in aquaculture were frequently detected. The tetracycline resistance gene tet (A), associated with oxytetracycline resistance, was identified in 78% of the isolates (7/9). Genes conferring resistance to quinolones, including *qnrS1* and *qnrVC1*, were detected in 67% of the genomes, while the sulfonamide resistance gene *sul1* was also present in 67% of the isolates.

Several AMR determinants relevant to human medicine were also identified. All genomes harbored β-lactam resistance genes belonging to the blaOXA family (9/9), indicating a widespread genetic potential for reduced susceptibility to β-lactam antibiotics. In addition, the aminoglycoside-modifying enzyme gene *aac6*′*)-Ib-cr*, which has been associated with reduced susceptibility to aminoglycosides and fluoroquinolones, was detected in 56% of the isolates. The carbapenemase gene *cphA* was identified in 33% of the genomes. This gene, which has been described as an intrinsic metallo-β-lactamase in *Aeromonas* species, was detected across multiple isolates, further expanding the diversity of AMR genes identified in the dataset.

Comparison of antimicrobial resistance genotypes and phenotypic susceptibility profiles revealed broad concordance across the analyzed isolates (Supplementary Table 4). Tetracycline-resistant isolates frequently harbored tet-family genes, whereas quinolone- and fluoroquinolone-resistant isolates carried qnr-associated determinants. Intrinsic β-lactamase genes, including blaOXA and cphA, were consistently identified across multiple *Aeromonas* species, supporting the widespread distribution of intrinsic β-lactam resistance mechanisms within the genus.

Screening of quinolone resistance-determining regions (QRDRs) identified amino acid substitutions at position 83 of *GyrA* (S83I) in multiple isolates exhibiting quinolone and fluoroquinolone resistance phenotypes. No canonical QRDR-associated substitutions were detected in *ParC*. These findings suggest that both plasmid-mediated quinolone resistance determinants and chromosomal QRDR mutations may contribute to the observed resistance profiles.

Overall, the resistome profiles revealed by genome analysis were broadly consistent with the antimicrobial susceptibility patterns observed among the *Aeromonas* isolates (Figure 4).

### 3.8 Predicted human pathogenic potential of Amazonian *Aeromonas* isolates

Prediction of human pathogenic potential using PathogenFinder v2 revealed consistently high pathogenicity scores across all nine Amazonian *Aeromonas* genomes analyzed. Mean pathogenicity probabilities ranged from 0.93 to 0.96, indicating the presence of genomic features associated with bacteria known to cause human infections, independent of species assignment or sequence type.

## 4 Discussion

This study provides the first genome-resolved characterization of *Aeromonas* spp. circulating in freshwater aquaculture systems in the Peruvian Amazon, and to our knowledge, represents the first whole-genome sequencing-based analysis of *Aeromonas* populations conducted in any Amazonian region of South America. By integrating whole-genome sequencing, phylogenomics, phenotypic antimicrobial susceptibility testing, and comparative genomic analyses, the analyzed isolates exhibited substantial genetic diversity and belonged to multiple phylogenetic lineages: *A. hydrophila, A. caviae*, and *A. veronii*. This genomic diversity provides critical baseline data for understanding *Aeromonas* evolution and dissemination in tropical aquaculture environments where clinical surveillance data remain limited.

Core genome phylogenomic reconstruction revealed that the isolates were distributed across multiple well-supported intra-species lineages rather than clustering according to host species or geographic origin. Comparable genome-scale studies of freshwater fish-associated *Aeromonas* populations have reported extensive clonal heterogeneity, with more than 120 sequence types and over 150 distinct phylogenetic clones identified within single geographic regions (Wang et al., 2025), underscoring the remarkable evolutionary plasticity of this genus in aquatic ecosystems. Similarly, investigations in ornamental fish have demonstrated the co-circulation of multiple intra-species subclades within confined aquatic systems (dos Santos et al., 2023), suggesting that ecological complexity rather than host specificity drives population structure. The absence of host-specific clustering further suggests ecological flexibility, consistent with the opportunistic nature of *Aeromonas* spp. in freshwater systems.

The multiclonal structure was further supported by rMLST analysis, which identified eight distinct ribosomal sequence types across the nine isolates, with only CAPA008 and CAPA022 sharing an identical rST (220374), suggesting potential clonal relatedness despite their recovery from independent aquaculture systems. Similar studies integrating WGS and MLST have reported extensive clonal heterogeneity across clinical, animal, and environmental *Aeromonas* isolates (Vieira et al., 2025), highlighting the remarkable genetic diversity of this genus. These findings underscore the need for expanded genomic surveillance of South American *Aeromonas* populations, which remain underrepresented in global molecular typing databases.

Comparative analysis of gene content across the *Aeromonas* genomes revealed substantial genomic diversity, with only a small proportion of genes shared across all isolates and a large fraction variably distributed among lineages. This pattern is consistent with previous genomic studies reporting high gene content variability within the genus (Ghatak et al., 2016; Abdella et al., 2023). Importantly, given the multi-species composition of the dataset, the set of genes shared across all genomes likely represents broadly conserved cellular functions rather than a biologically meaningful core genome. Conversely, the variable gene repertoire reflects lineage-specific gene content differences that may contribute to ecological adaptation and functional diversity across *Aeromonas* populations. In this context, the present analysis should be interpreted as a comparative framework of gene content variation rather than a formal characterization of pangenome structure.

Comparative genomic evidence supports the context-dependent and lineage-specific nature of *Aeromonas* virulence. In Amazonian aquaculture, molecular characterization of isolates from diseased *Piaractus brachypomus* revealed widespread distribution of structural virulence genes (flagella, adhesins) while classical enterotoxins (*aerA* and *act*) were detected at lower frequencies, with mortality varying despite distinct gene profiles (Medina-Morillo et al., 2023). In contrast, genome-based investigations of *Aeromonas* associated with motile septicemia in Southeast Asia demonstrated that epidemic lineages frequently harbored enterotoxins and aerolysin, yet structural determinants such as flagella and secretion systems were conserved across species (Truong et al., 2024). The Amazonian isolates analyzed here lack major cytotoxic enterotoxins, suggesting a virulence architecture emphasizing colonization capacity (flagella, MSHA pilus), Type II/VI secretion systems, and environmental fitness rather than acute toxin-mediated pathogenicity. This pattern is consistent with environmental opportunistic pathogens rather than acute epidemic lineages. Collectively, these findings reinforce the view that *Aeromonas* virulence is multifactorial, lineage-dependent, and shaped by ecological context rather than defined by a uniform set of toxin genes.

Consistent with this interpretation, *in silico* prediction of human pathogenic potential using PathogenFinder v2 yielded uniformly high probability scores (0.93–0.96) across all isolates, irrespective of species or sequence type. Although canonical enterotoxin genes (*aerA, act*, and *ast*) were absent, these predictions indicate that the broader genomic background of the isolates shares similarity with strains documented to cause human infections. This apparent paradox—high pathogenicity predictions despite the absence of major toxins—highlights the multifactorial nature of *Aeromonas* pathogenesis.

Importantly, PathogenFinder V2 predictions are based on genome-wide similarity patterns to known human pathogens and therefore do not rely exclusively on the presence of classical toxin genes. In this context, conserved secretion-associated systems, quorum sensing pathways, colonization-related structures, and lineage-specific accessory genes may collectively contribute to the predicted opportunistic pathogenic potential observed across the analyzed genomes. Rather than relying on acute toxin determinants, these isolates likely exhibit pathogenic potential through synergistic effects of: (i) conserved colonization factors (flagella, MSHA pilus, type IV pili); (ii) Type II and Type VI secretion systems for protein delivery; and (iii) lineage-specific accessory genes acquired through horizontal gene transfer. Such architecture is consistent with emerging evidence indicating that environmental *Aeromonas* strains frequently cause opportunistic infections in immunocompromised hosts through mechanisms distinct from classical profiles observed in epidemic aquaculture strains. This observation reinforces the necessity for genomic surveillance to predict human health risks from aquaculture-derived pathogens.

The integration of phenotypic antimicrobial susceptibility testing and whole-genome resistome analysis revealed multiple antimicrobial resistance determinants associated with compounds widely used in aquaculture. The high prevalence of *tet (A)* (78%) aligns with extensive tetracycline use in fish production systems and is consistent with large-scale comparative genomic analyses documenting global dissemination of tetracycline resistance genes in aquaculture-associated *Aeromonas* populations (Roh and Kannimuthu, 2023). In global datasets, approximately 25% of *Aeromonas* strains harbor *tet (A), tet (D)*, or *sul1*; the markedly higher prevalence in the present Amazonian isolates (78% *tet (A)*, 67% *sul1*) likely reflects localized or regional antimicrobial selection pressures. The detection of plasmid-mediated quinolone resistance genes (*qnrS1* and *qnrVC1*) in 67% of isolates further supports sustained antimicrobial exposure, positioning these isolates within the expanding global resistome shaped by aquaculture practices.

All isolates harbored β-lactam resistance genes of the *blaOXA* family, reflecting the intrinsic reduced susceptibility of *Aeromonas* spp. to β-lactams. Notably, one-third of Amazonian isolates (33%, *n* = 3) also carried *cphA*, a carbapenemase gene encoding an intrinsic metallo-β-lactamase. Comparative genomic analyses across multiple *Aeromonas* species have similarly documented widespread distribution of *cphA* and OXA-family β-lactamases within the genus (Maia et al., 2023). The detection of *cphA* in the present isolates reinforces the role of *Aeromonas* as a natural environmental reservoir of carbapenemase-related determinants.

Although no acquired high-priority carbapenemases (e.g., blaNDM-1) were detected in this study, recent clinical reports documenting NDM-1-producing *Aeromonas caviae* highlight the evolutionary capacity of this genus to acquire critical resistance genes under selective pressure (Hu et al., 2023). This observation underscores the strategic importance of genomic surveillance of aquaculture-associated populations before such determinants emerge regionally.

Resistome profiles were broadly concordant with phenotypic susceptibility data, supporting the reliability of genome-based AMR prediction in environmental isolates. However, as the genomic context and mobility of resistance genes were not specifically investigated, the extent to which these determinants may disseminate via horizontal gene transfer within aquaculture systems remains to be determined.

The identification of virulence and antimicrobial resistance genes in this study was performed entirely *in silico*. Although no experimental validation of individual genes was conducted, the observed concordance between predicted resistance determinants and phenotypic antimicrobial susceptibility profiles supports the robustness of the genomic approach for AMR inference. Nevertheless, functional validation would be required to confirm the expression and activity of specific virulence and resistance genes.

The absence of classical toxin genes such as *aerA, act*, and *ast* does not preclude pathogenic potential in *Aeromonas* spp. Pathogenesis in this genus is widely recognized as multifactorial and not dependent on a single set of toxin determinants. Instead, virulence may arise from the combined action of colonization factors (e.g., flagella, pili), secretion systems, metabolic adaptability, and accessory genes that enhance host interaction and environmental persistence. In this context, the high pathogenicity scores predicted *in silico* likely reflect the overall genomic background shared with clinically relevant strains rather than the presence of specific toxin genes alone. This highlights the importance of considering genome-wide virulence architectures rather than relying solely on canonical markers.

In addition, the detection of multiple antimicrobial resistance genes raises concerns regarding their potential dissemination within aquaculture environments. *Aeromonas* spp. are known to harbor mobile genetic elements, including plasmids, integrons, and transposons, which facilitate horizontal gene transfer (HGT) between environmental and pathogenic bacteria. In densely populated aquaculture systems, where antimicrobial use and environmental stressors coexist, such conditions may promote the exchange of resistance determinants across bacterial communities. This process could contribute to the emergence of multidrug-resistant strains with implications for fish health, environmental microbiomes, and human infections, particularly in settings where water systems interface directly with human populations. These findings reinforce the relevance of a One Health perspective in understanding the ecological and public health risks associated with antimicrobial resistance in aquatic environments.

Collectively, these results indicate that Amazonian freshwater aquaculture environments may function as regional reservoirs of globally distributed antimicrobial resistance determinants, reinforcing the need for prudent antimicrobial stewardship and sustained genomic surveillance within a One Health framework.

The combined phylogenomic, virulence, and resistome analyses presented in this study underscore the critical importance of Amazonian aquaculture systems within an integrated One Health framework. The detection of genetically diverse *Aeromonas* lineages harboring conserved pathogenic determinants and multiple clinically relevant antimicrobial resistance genes reveals that these freshwater systems function as dynamic ecological interfaces connecting environmental, animal, and human-associated microbial reservoirs (Milijasevic et al., 2024). In the Peruvian Amazon—where aquaculture operations are embedded within riverine ecosystems in close proximity to indigenous and rural communities—such ecological connectivity may facilitate bidirectional exchange of bacterial strains and resistance determinants. Aquaculture workers handling diseased fish, consumers of undercooked aquatic products, and immunocompromised individuals in surrounding communities may be at particular risk for infections caused by these environmental strains. Integration of genomic monitoring into aquaculture health management, coupled with coordinated surveillance linking veterinary, environmental, and public health sectors, is therefore essential to prevent the emergence and dissemination of clinically consequential *Aeromonas* lineages.

Although the isolates analyzed were obtained from fish, the consistently high predicted human pathogenicity scores and the presence of clinically relevant antimicrobial resistance genes underscore the potential cross-sectoral relevance of these lineages for human health. *Aeromonas* spp. are increasingly recognized as environmentally adapted opportunistic pathogens with the capacity to bridge aquatic and human-associated niches, and large-scale genomic analyses have demonstrated extensive diversity, frequent antimicrobial resistance gene carriage, and the absence of strict separation between clinical and environmental strains (Singh et al., 2026). These observations reinforce the evidence that aquaculture environments may serve as regional reservoirs or amplification platforms for *Aeromonas* lineages with zoonotic and public health significance.

Notably, South American and tropical freshwater systems remain substantially underrepresented in global genomic surveillance datasets, fundamentally limiting our understanding of *Aeromonas* evolutionary dynamics and antimicrobial resistance dissemination patterns in these ecologically and economically critical regions (David et al., 2025). Expanding genomic representation from the Amazon basin is therefore strategically essential to achieve a more geographically balanced and epidemiologically informative view of *Aeromonas* diversity at the human–animal–environment interface, and to develop region-specific predictions of pathogenic potential in emerging lineages.

Taken together, these results support the integration of genomic monitoring into aquaculture health management and emphasize the importance of coordinated surveillance strategies linking veterinary, environmental, and public health sectors under a One Health framework.

A key limitation of this study is that gene presence–absence analyses were conducted across multiple *Aeromonas* species. As increasing taxonomic diversity leads to a reduction in the number of shared genes, species-specific core gene sets may be underrepresented in this framework. Species-level pangenome analyses, incorporating larger collections of high-quality genomes for individual *Aeromonas* species, would likely reveal more extensive and biologically informative core genomes, including lineage-specific conserved functions. Additionally, future studies integrating the characterization of mobile genetic elements, such as plasmids, integrons, and transposons, will be essential to better understand the genomic context and dissemination potential of antimicrobial resistance genes within *Aeromonas* populations, particularly in aquaculture systems under a One Health framework.

## 5 Conclusion

This study provides the first genome-resolved characterization of *Aeromonas* spp. circulating in freshwater aquaculture systems in the Peruvian Amazon, revealing substantial genetic diversity among isolates belonging to *A. hydrophila, A. caviae*, and *A. veronii*. Despite the absence of major cytotoxic toxin genes, the isolates shared conserved colonization- and secretion-associated determinants and exhibited high predicted human pathogenic potential. Multiple antimicrobial resistance determinants relevant to both aquaculture and clinical contexts were identified, including plasmid-mediated quinolone resistance genes and QRDR-associated mutations, with genomic profiles largely concordant with phenotypic susceptibility data.

Collectively, these findings underscore the ecological complexity and public health relevance of aquaculture environments in Peru as reservoirs of opportunistic *Aeromonas* lineages. While gene content analyses highlighted extensive genomic variability across the dataset, they are interpreted within a comparative framework rather than as a formal characterization of pangenome structure. Our results highlight the importance of integrating genomic surveillance into aquaculture health management and reinforce the need for coordinated monitoring strategies across environmental, animal, and human sectors within a One Health framework. Future studies incorporating species-level genomic analyses and the characterization of mobile genetic elements will be essential to further elucidate the dynamics of antimicrobial resistance dissemination in *Aeromonas* populations.

## Data Availability

The raw data generated in this study can be found in the NCBI GenBank (https://www.ncbi.nlm.nih.gov/genbank), accession PRJNA902004.
