# Supplementary material for: Phylogenomic and comparative genomic analyses of Aeromonas spp. from South American aquatic systems reveal extensive genomic diversity, antimicrobial resistance, and predicted human pathogenicity
Source: Front Microbiol. 2026 Jun 16;17:1823138. doi: 10.3389/fmicb.2026.1823138 (PMC13317488; doi:10.3389/fmicb.2026.1823138)

## ****Supplementary Figure S1.** Average Nucleotide Identity (ANI) analysis of** Aeromonas **genomes**

(A) Pairwise ANI heatmap of the 112 Aeromonas genomes analyzed in this study, illustrating genome-wide similarity and species-level clustering patterns. (B–D) Within-species ANI heatmaps showing fine-scale genomic relatedness for (B) Aeromonas hydrophila, (C) Aeromonas caviae, and (D) Aeromonas veronii. ANI values are expressed as percentage identity. A threshold of ≥95% ANI was used as a criterion for species delineation.


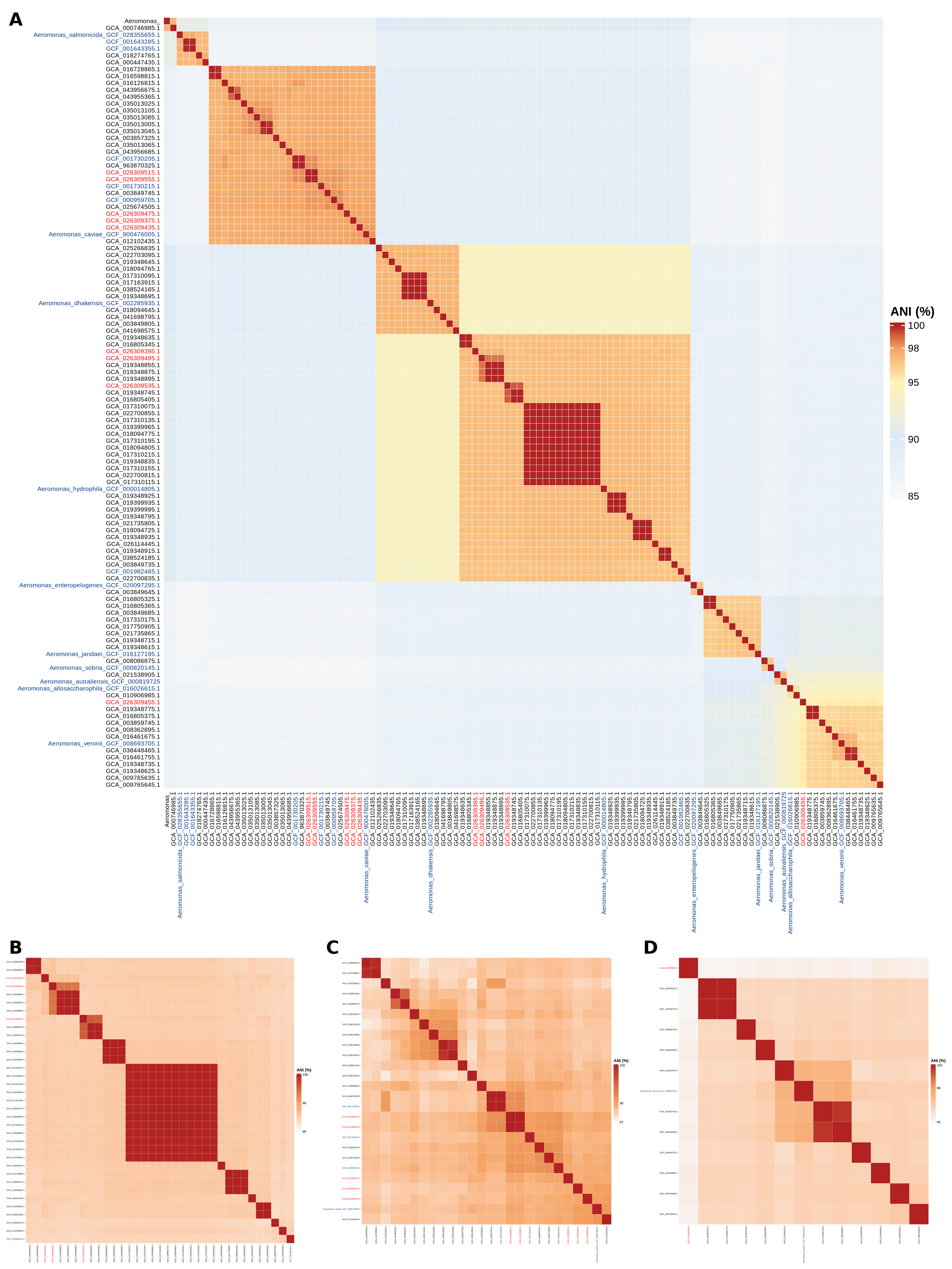

Supplement: Supplementary file 1 [file Data_Sheet_1.docx]
